# Disentangling the Influence of Environment, Host Specificity and Thallus Differentiation on Bacterial Communities in Siphonous Green Seaweeds

**DOI:** 10.3389/fmicb.2019.00717

**Published:** 2019-04-05

**Authors:** Kathryn Lee Morrissey, Levent Çavaş, Anne Willems, Olivier De Clerck

**Affiliations:** ^1^Department of Biology, Phycology Research Group, Ghent University, Ghent, Belgium; ^2^Department of Chemistry, Biochemistry Division, Faculty of Science, Dokuz Eylül University, İzmir, Turkey; ^3^Laboratory of Microbiology, Department of Biochemistry and Microbiology, Ghent University, Ghent, Belgium

**Keywords:** *Caulerpa*, microbiome, bacterial variation, host specificity, morphological niche

## Abstract

Siphonous green seaweeds, such as *Caulerpa*, are among the most morphologically complex algae with differentiated algal structures (morphological niches). *Caulerpa* is also host to a rich diversity of bacterial endo- and epibionts. The degree to which these bacterial communities are species-, or even niche-specific remains largely unknown. To address this, we investigated the diversity of bacteria associated to different morphological niches of both native and invasive species of *Caulerpa* from different geographic locations along the Turkish coastline of the Aegean sea. Associated bacteria were identified using the 16S rDNA marker gene for three morphological niches, such as the endobiome, epibiome, and rhizobiome. Bacterial community structure was explored and deterministic factors behind bacterial variation were investigated. Of the total variation, only 21.5% could be explained. Pronounced differences in bacterial community composition were observed and variation was partly explained by a combination of host species, biogeography and nutrient levels. The majority of the explained bacterial variation within the algal holobiont was attributed to the micro-environments established by distinct morphological niches. This study further supports the hypothesis that the bacterial assembly is largely stochastic in nature and bacterial community structure is most likely linked to functional genes rather than taxonomy.

## Introduction

Bacteria are omnipresent in the marine environment. Biologists have also become increasingly aware that some bacteria form close associations with eukaryotic organisms (Relman, 2008). Essential symbiotic relationships are established between bacteria and host organisms such as corals, sponges, and macroalgae. Both corals and sponges are host to a rich diversity of microbes that are integral to their health and ecological success (Rosenberg et al., 2007; Webster and Thomas, 2016). In seaweeds bacteria have been identified to associate both as intracellular endobionts and as epibionts integrated in complex surface biofilms (Egan et al., 2013). Endobionts and epibionts are distinct from one other and are hypothesized to contribute independently to specific functions that aid in maintaining regular functioning of the host (Wahl et al., 2012; Singh and Reddy, 2014). Certain bacteria have developed functionally integrated symbioses with their respective host species and therefore can be regarded as a single biological entity, or holobiont. The genes present in the host as well as the associated microbial component are therefore collectively referred to as the hologenome (Reshef et al., 2006; Zilber-Rosenberg and Rosenberg, 2008). Further reinforcing the hologenome theory, is the presence of cooperative bacteria that produce essential secondary metabolites and nutrients (Chisholm et al., 1996; Dobretsov et al., 2006), which have been directly and indirectly linked to growth, morphogenesis and the ecological success of macroalgae (Aires et al., 2013; Hollants et al., 2013). Specific extracellular metabolites from the Cytophaga-Flavobacterium-Bacteroides group have been identified to have a direct influence on differentiation of *Monostroma oxyspermum*, as well as the normal morphogenesis and germination of *Ulva* species (Matsuo et al., 2003; Spoerner et al., 2012). The algal chemosphere accommodates a complex interaction of cross-kingdom signaling molecules to facilitate symbiotic growth and development. For example, *Ulva* produces a mix of chemicals including DMSP, involved in microbial gardening that attracts and promotes the growth of beneficial bacteria. These symbiotic bacteria in turn release a range of metabolites, known as morphogens, that mediate successful cell division and rhizoid formation in the alga (Kessler et al., 2018).

Despite these specific interactions high variability is observed in bacterial communities associated to macroalgae (Singh and Reddy, 2014), which suggests that stochastic processes are also involved in their assembly and recruitment. While variability at the individual level of host-associated bacteria is seemingly universal from observations regarding the human microbiome to observations in marine systems, these communities exhibit functional redundancy (Burke et al., 2011b; Bashan et al., 2016). Burke et al. (2011a) therefore proposed a lottery hypothesis illustrating that the assembly of specific bacterial groups in algae is related to their functional diversity as opposed to their taxonomic structure. Under this lottery hypothesis, the functional competency of a bacterial species is the key to their recruitment by a host. This has been recently demonstrated for *Ulva australis* where a core bacterial community could not be identified from the taxonomic composition, but rather a functional core was defined based on common gene profiles (Roth-Schulze et al., 2018).

**FIGURE 1:**
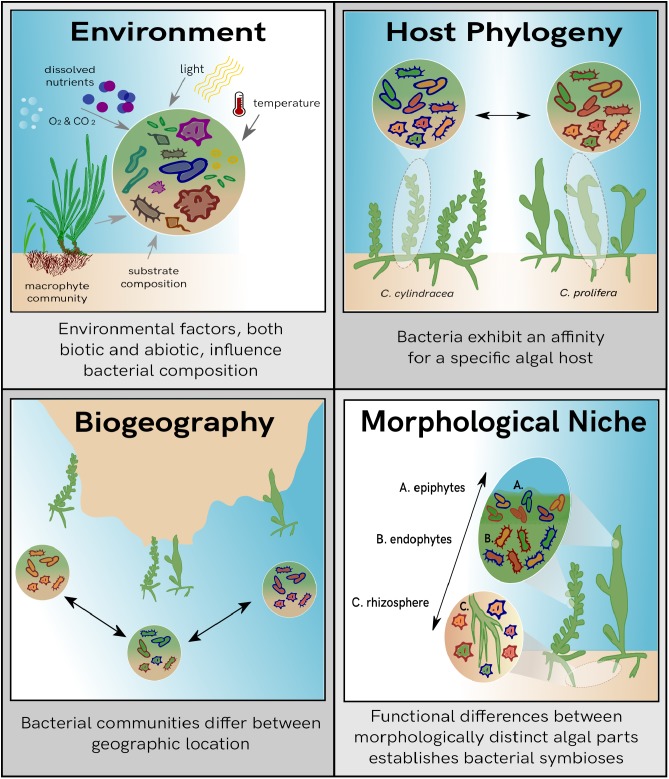
Overview of the potential influences on bacterial community composition. Each panel describes individual drivers of bacterial variation observed within the marine biosphere.

Several potential factors are involved in shaping bacterial communities and can act either independently of one another or as a combination of multiple effects (Figure 1). Environmental factors dictate the functional requirements of an ecosystem (Darling and Cote, 2008), and therefore are key drivers in bacterial community composition (Egan et al., 2013). Additionally, the evolutionary history of the host influences associated bacterial composition, with certain bacterial groups showing a higher affinity toward specific host species (Hollants et al., 2011). Bacterial community composition can also be attributed to biogeography, although its contribution has been shown to be less notable than the variation seen from environmental factors (Lima-Mendez et al., 2018). While biogeography mainly dictates the range of biological dispersal, it can influence both available hosts as well as environmental parameters such as nutrient profiles, temperature, benthic habitat, and light availability. Therefore, combining all these factors together, a biogeographic signature can be seen for bacterial community profiles. Although a large homogeneity of bacterial species is still observed across geographic regions and the signal is less prominent than what is seen for macro-organisms, environmental parameters should still be considered a driving force behind observed bacterial structure (Van der Gucht et al., 2007).

The importance of bacterial interactions in regard to seaweeds is evident through the study of several organisms. More specifically siphonal green algae, which are large single-celled organisms with multiple nuclei that are differentiated into morphologically distinct niches (Jacobs, 1994), have been shown to host integrated bacteria, both as endobionts and epibionts (Delbridge et al., 2004; Aires et al., 2013). Taxonomically diverse bacterial communities have been shown in *Bryopsis*, with certain bacterial groups consistently identified across geographical regions (Hollants et al., 2011). The green alga *Caulerpa* has also been shown to contain complex and rich bacterial associations and is emerging as a model to study algal-bacterial associations in green seaweeds (Aires et al., 2015; Arnaud-Haond et al., 2017). A high percentage of the endobacteria associated to *Caulerpa* show a tight association with their host species, while epibacterial communities are more likely to be influenced by the environment (Aires et al., 2013). A significant portion of these tightly associated endobionts has been suggested to be inherited by host progeny (Arnaud-Haond et al., 2017). This is consistent with findings from Burr and West (1970), where intracellular bacteria were observed inside the gametes from *Bryopsis hypnoides*. Several *Caulerpa* species from different geographic locations also share common bacteria (Aires et al., 2015), suggesting that some bacterial groups persist in various ecological niches regardless of host species or geography. Furthermore, differences identified in the transcriptome of *Caulerpa* from base to apex of the thallus (Coneva and Chitwood, 2015) suggests that functional requirements within siphonous algae are unique to each segment of the thallus. It has been described that differentiated parts of the siphonal algae *Caulerpa* form individual morphological niches that have distinct functions associated to each niche (Chisholm et al., 1996; Dobretsov et al., 2006; Ranjan et al., 2015), therefore it is logical to suggest that bacterial associations differ correspondingly.

Comparisons between the bacterial communities of the *Caulerpa* endobiome, epibiome, and rhizobiome have not yet been fully described, with little known regarding the environmental drivers of algal microbiome composition as well as these influences on particular morphological niches. In this study bacterial communities for different morphological niches of *Caulerpa cylindracea* and *Caulerpa prolifera* found along the Turkish coastline of the Aegean Sea were characterized. This study aims to (1) discriminate between bacteria associated to the endobiome, epibiome, and rhizobiome, (2) assess common and unique bacterial operational taxonomic units (OTUs) for each sample type, (3) partition driving forces behind bacterial community compositions, such as morphological niche, and a combination of host species and ecological factors, and (4) investigate drivers for each morphological niche individually. Here we propose two main hypotheses, firstly that bacterial species in common between algal hosts are also shared across morphological niches, whereas unique bacteria associated to a morphological niche are also then unique to each host species. Secondly we hypothesize that inter-variability within an algal thallus, i.e., functional requirements of each morphological niche, has a larger influence on bacterial community composition, than other environmental factors.

## Materials and Methods

### Sample Collection and Preparation

**FIGURE 2:**
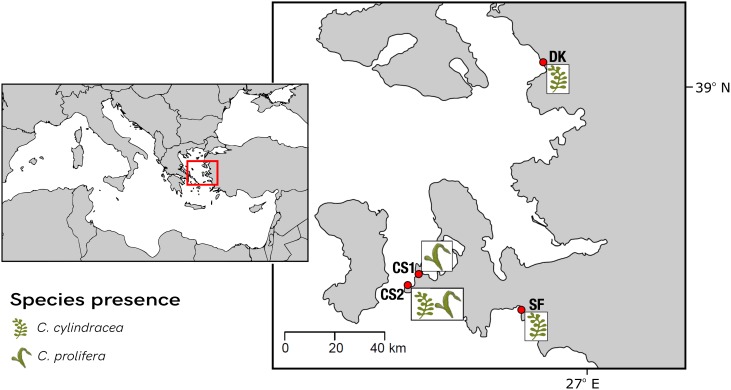
Map of the sampling sites along the İzmir coast in Turkey. A total of four sites were selected, two within the Çeşme region (CS1 and CS2) as well as Dikili (DK) and, Seferihisar (SF).

Along the İzmir coast of Turkey, four sampling sites were chosen for this study (Figure 2). Sites differed in their benthic composition, nutrient profiles and depth. Site characteristics collected *in situ* on day of sampling are summarized in Table 1. Native *C. prolifera* was present at two of the sites (CS1 and CS2), whereas the invasive *C. cylindracea* was present at three (SF, DK, CS2), with only one site, CS2, where both occurred together. Morphologically, sampling units (SUs) comprised of interconnected thalli, stolon, and rhizoids, were taken for each species from each site in triplicate, totaling 15 individuals. Each SU obtained originated from a separate algal individual. Each algal thallus was further separated into three distinct morphological niches, namely endobiome, epibiome, and rhizobiome fractions (*n* = 45). One thallus from each SU was surface sterilized using a protocol adapted from Hollants et al. (2013), in order to recover the endobiome fraction. Prior to sterilization, swabs of the surface epibionts were taken. Rhizoids were washed with sterile seawater and separated with a sterile blade. All samples were then frozen at -20°C until further analysis. Nutrient levels of nitrite, nitrate, phosphate, ammonia, and silicate were measured using a SAN++ Automated Wet Chemistry Analyzer – Continuous Flow Analyzer (Skalar Analytical B.V.) following the manufacturer’s instructions.

**Table 1:** Summary of the environmental parameters for each site.

Site	Substrate composition	Spatial coordinates	*Caulerpa* species	Sampling units (SU)	Total nr. samples	Temp (°C)	Nitrite (μg/l)	Nitrate (μg/l)	Silicate (μg/l)	Ammonia (μg/l)	Phosphate (μg/l)
SF	Mixed	38°11′50.5″N; 26°46′19.7″E	*C. cylindracea*	3	9	19	3.99	23.27	318.54	185.67	12.58
DK	Sandy	39°07′27.7″N; 26°51′08.1′E	*C. cylindracea*	3	9	18	5.3	28.01	49.99	367.49	64.17
CS1	Sandy	38°19′39.6″N; 26°17′50.3″E	*C. prolifera*	3	9	17	1.17	15.29	49.99	50	0.99
CS2	Sandy	38°19′03.5″N; 26°17′12.0″E	*C. cylindracea* and *C. prolifera*	6	18	19	1.18	7.87	49.99	75.99	0.9


### DNA Isolation and Sequencing

Microbial DNA was extracted from all samples following the protocol by Doyle and Doyle (1987), with slight modifications. Both the thallus and rhizoid samples were homogenized with liquid nitrogen and were subjected to an additional bead-beating step preceding the standard protocol. The bacteria-specific 16S rDNA gene was amplified in a nested PCR approach, first using the universal primers 27F (5′-AGAGTTTGATCMTGGCTCAG-3′) and 1492R (5′-TACGGYTACCTTGTTACGACTT-3′) according to Lane (1991). A post-PCR cleanup was done using AMPure^®^ bead purification (Beckman Coulter, Inc., CA, United States) according to the manufacturer’s instructions. Following the approach used by Aires et al. (2012), a second PCR was done targeting the V5–V7 hypervariable regions of the bacterial 16S rRNA gene using the primers 799mod3F (5′-GGATTAGATACCKGG-3′) and 1193R (5′-ACGTCATCCCCACCTTCC-3′) which have been shown by Hanshew et al. (2013) and Bodenhausen et al. (2013), to reduce chloroplast contamination. These primers included Illumina overhang sequences used for ligation of index tags during library preparation. The second PCR started with an initial denaturation step of 5 min at 95°C. This step was followed with 10 cycles of 1 min denaturation at 95°C, a touchdown annealing step starting at 65°C, decreasing in increments of 0.5 to 60°C and an extension at 72°C for 3 min. An additional 15 cycles using the lowest annealing temperature were then performed, with a final extension period of 20 min at 72°C. Again AMPure^®^ bead purification was used to clean PCR products. Sequence libraries were constructed using the NexteraXT DNA sample preparation kit for dual indexing of the sequences, ligated by an additional PCR of 12 cycles. Sequences were sent to BaseClear for Illumina MiSeq v3 (2 × 300 bp) sequencing after additional AMPure^®^ bead purification and equimolar pooling of the samples.

### Data Processing

Demultiplexed files with primers pre-trimmed were obtained from the sequencers. After initial quality assessment of the raw amplicon reads, the reverse reads were trimmed to improve overall sequence quality. Paired-end sequences were then merged using the BBMerge function as a part of the BBTools package (Bushnell et al., 2017) with a minimum overlap of 50 bp and no gaps were allowed in the overlapping region of the aligned reads. Reads with a length less than 260 were removed before assembly. Further quality filtering was done with a maximum expected error set at 0.5 and assembled reads with a length longer than 420 bp were discarded. Following the pre-processing of the reads, sequences were processed using a the UPARSE pipeline (Edgar, 2013) as part of the USEARCH package (Edgar, 2010). After dereplication OTUs were binned at a similarity threshold of 97%. Clustering was done using the *cluster_otus* command based on the UPARSE-OTU algorithm which simultaneously removes chimeric sequences. Taxonomy was assigned with mothur (Schloss et al., 2009) using the RDP database release 11 (Cole et al., 2014) at a confidence of 90%. An OTU table was generated and amended with metadata using qiime 2 (Caporaso et al., 2010) in the biom JSON format for further statistical analysis.

### Statistical Analyses and Visualization

All statistical analyses were performed in R software 3.1.2 (R Development Core Team, 2010), using the “phyloseq” package (Mcmurdie and Holmes, 2013). Data were imported as a biom complex file incorporating a phylogenetic tree constructed using FastTree2 (Price et al., 2010). All samples were rarefied to the minimum sample size of 3753 reads, with a Good’s coverage index calculated for each sample (Good, 1953). Sample β-diversity was explored visually using a non-metric multidimensional scaling (NMDS) plot using Bray-Curtis dissimilarity and variables were investigated by permutational multivariate analysis of variance (PERMANOVA) performed using the *adonis2* function in the “vegan” R-package version 2.4-6 (Oksanen et al., 2016). Moreover, variation partitioning was done using the *varpart* function also included in the “vegan” R-package version 2.4-6 (Oksanen et al., 2016) and relevant explanatory matrices tested for significance using the *anova.cca* command. For these analyses, nutrient values were log transformed and spatial coordinates were converted into Principal Coordinates of Neighborhood Matrix using distances calculated in the *earth.dist* command as part of the “fossil” R-package. Further community comparisons were explored using the “microbiome” R-package (Lahti and Shetty, 2017) and key bacteria, classified to order level, were identified by performing a species indicator analysis with the “indispecies” R-package, v 1.7.6 (De Caceres and Legendre, 2009).

## Results

**FIGURE 3:**
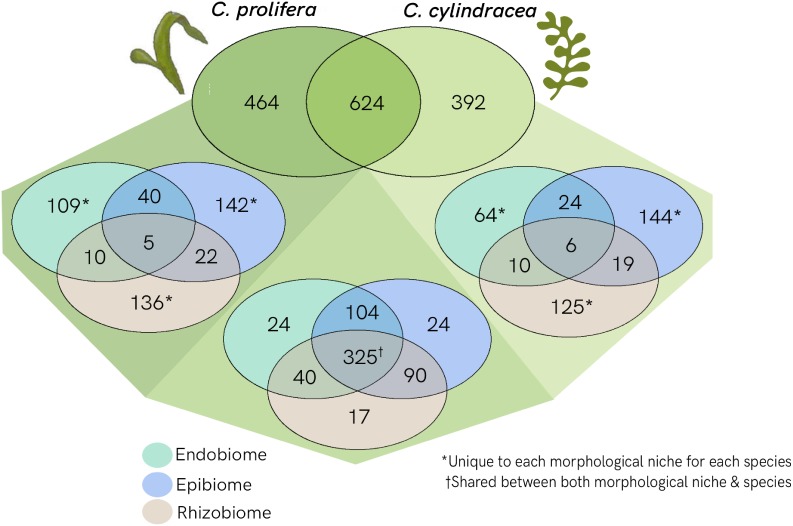
Venn diagram showing the separation of OTUs between *Caulerpa* species and morphological niches. Shared OTUs as well as unique OTUs for each species are shown in shades of green. Further separations between morphological niches for both shared and unique OTUs are shown: endobiome (green); epibiome (blue); rhizobiome (beige).

Applying a metabarcoding approach targeting the V5–V7 region of the 16S rRNA gene, a total of 1,05 million raw paired–end reads (approximate length of 300 bp each) were generated using the Illumina MiSeq v3 platform. After merge-pair assembly, chimera detection and quality filtering, 86% of the reads were retained. Downstream analysis was performed on 907,639 high quality reads corresponding to the sample set used in this study. The OTU richness observed in the samples was 1657, with OTU binning set at 97% similarity. The number of OTUs per sample ranged from 62 to 469 OTUs, with an average of 14,516 reads per sample and a Good’s coverage between 98.5 and 99.9% (Supplementary Table S1). Read numbers were normalized through rarefaction to the minimum number of reads, 3640 (Supplementary Figure S1), removing 177 OTUs of low abundance. A total of 1480 OTUs remained after rarefaction (Figure 3), of which 624 were shared between both *Caulerpa* species. *C. prolifera* had a set of 464 unique OTUs, whereas *C. cylindracea* had 392 unique OTUs. Of the shared fraction, 325 OTUs were found across all three morphological niches. Within the species-specific OTUs, a higher number of OTUs were found to be unique to each morphological niche as opposed to being shared among the niches.

**FIGURE 4:**
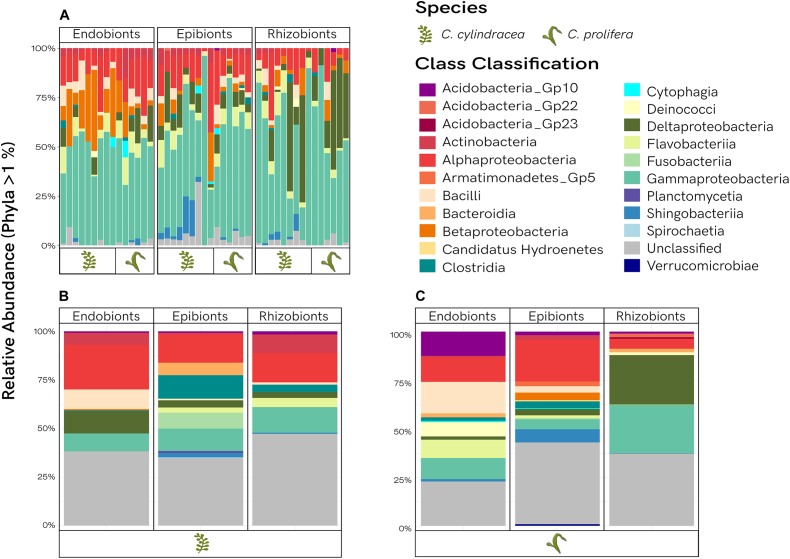
Taxanomic distribution of the bacterial communities. Relative abundances of phyla are shown for the shared fractions for each *Caulerpa* species **(A)**, as well as the average relative abundances of unique fractions for *C. cylindracea* **(B)**, and *C. prolifera* **(C)**.

Bacterial communities shared between *Caulerpa* species are presented in Figure 4A. Classification is comprised of 13 classes, with the unclassified portion constituting less than 10% on average. The number of Proteobacteria, dominated by Gammaproteobacteria, outweighed the other taxonomic groups across all morphological niches. Betaproteobacteria were more prevalent in the endobiome, whereas Deltaproteobacteria were more abundant in the rhizobiome. Alphaproteobacteria were found consistently across all samples. Although variability is observed between samples, overall the community structure of each morphological niche among the shared OTUs was noticeably similar. The classification of the unique OTUs specific to each *Caulerpa* species is illustrated by Figure 4B,C, with the relative abundances averaged across samples to observe trends. Proteobacteria were still found in high abundances and were dominant in all niches, but class structure within this phylum varied among sample types. In contrast to the shared fraction, community profiles of the unique OTUs were distinct from each other, with more unclassified phyla observed (∼18–40%). Within these unique fractions, Fusobacteriia were only present in the epibiont samples, with high abundances observed in *C. cylindracea*, and minimal presence in *C. prolifera*. Comparing the unique OTUs for the two species, Actinobacteria are shown to be more abundant than Acidobacteria *Gp10* in *C. cylindracea*, however, the opposite is true for *C. prolifer*a. Bacteroidia were found in the unique endobiome and rhizobiome fractions for *C. prolifera*, as well as the unique epibiome fraction of *C. cylindracea*. Bacilli were more abundant in both endobiomes. Planctomycetia were only present in the unique endobiome and epibiome *C. cylindracea* OTUs. Verrucomicrobia were exclusively observed in the unique endobiome and epibiome OTUs of *C. prolifera*, with Armatimonadetes Gp5 also exclusively being found in the epibiome fraction.

Comparisons between microbiomes were done to analyze significant factors influencing the bacterial composition. A NMDS plot was done to visually investigate the β-diversity of the samples (Supplementary Figure S2). No highly distinct separations were observed and the potential explanatory variables were further investigated using PERMANOVA. Results of the PERMANOVA (Table 2 and Supplementary Table S2) indicate that morphological niche (*p* = 0.0001), environmental conditions (*p* = 0.0001) and the combination thereof (*p* = 0.0211) contributed significantly to the differences observed. Furthermore, host species had a significant effect on the bacterial communities (*p* = 0.0222). Different factors were observed to influence the bacterial diversity for the individual morphological niches (Table 2 and Supplementary Table S2). While the influence of both host species and the environment was significant for the epibiome (*p* = 0.037 and *p* = 0.0079, respectively), neither showed a significance for the endobiome. The bacterial rhizobiome demonstrated significant influences from environmental factors (*p* = 0.0431) alone.

**Table 2:** Summary of the PERMANOVA analyses based on Bray–Curtis dissimilarities of OTU abundances for bacterial communities within the whole microbiome and separate morphological niches.

Factor	Complete microbiome	Endobiome	Epibiome	Rhizobiome
Morphological niche	**0.0001**	–	–	–
Host	**0.0222**	0.0501	**0.037**	0.2437
Environment^∗^	**0.0001**	0.2074	**0.0079**	**0.0431**
Morphological niche:host	0.0753	-	–	–
Morphological niche:environment^∗^	**0.0211**	–	–	–


*^∗^Environment = spatial coordinates + nutrient profiles; Significant *p*-values (alpha ≤0.05) are shown in bold.*

To further investigate the drivers of bacterial diversity between samples, variation partitioning was performed. Explanatory variables with a significant influence on total variation were morphological niche (*p* = 0.002) and a combination of environmental factors (*p* = 0.047), including physiochemical parameters, host species and spatial coordinates (Table 1). While the majority of the variation observed remains unexplained at 78.50%, the morphological niche exclusively contributes to 12.35% of the variation, while the environmental component is responsible for 8.02% of the variation. Additionally, when examining the influences on individual bacterial phyla, proportions attributed to the explanatory variables shift depending on the phylum. Significant contributing factors for each phylum were summarized (Table 3). The contribution patterns observed for the entire dataset are also observed for the most dominant phylum, Proteobacteria. Analysis of Bacteroidetes and Acidobacteria revealed less than 5% of the variation can be explained by either explanatory variable. For Actinobacteria, morphological niche influences 7.01% of the variation, whereas the environment contributes less than 1% to the variation. Alternatively, when focusing on Ignavibacteriae, the variation observed is exclusively attributed to the environment (6.86%).

**Table 3:** Significant contributions to observed variation for individual bacterial phyla for all three morphological niches.

Phylum	Source of variation					
	
	Unexplained	Morphological niche	*p*-value	Environment^∗^	Contributing environmental factor	*p*-value
*Actinobacteria*	92.11%	7.01%	0.007	0.15%	-	
*Acidobacteria*	97.13%	0.78%	0.014	1.97%	Host	0.0002
					Ammonia	0.0030
*Bacteroidetes*	95.00%	1.24%	0.507	3.54%	Silicate	0.0033
					Nitrite	0.0549
*Ignavibacteria*	93.14%	0.00%	0.177	6.86%	Silicate	0.0080
*Proteobacteria*	77.17%	13.97%	0.001	7.24%	Nitrite	0.0357
					Silicate	0.0744


*^∗^Combination of all environmental variables (geography, host, nutrient levels and temperature).*

Separating the individual morphological niches, provided no significant partitioning for the bacterial variation in the endobiome and epibiome. However, when analyzing the rhizobiome separately, the contribution of environmental influence is 30.48% (*p* = 0.042), with 69.52% remaining unexplained. The factor contributing significantly to the environmental influence is nitrate level (*p* = 0.0457). Further analysis for each bacterial phyla indicated the significant influence of environmental factors for four phyla, Acidobacteria, Actinobacteria, Deinococcus-Thermus, and Proteobacteria. For Proteobacteria and Actinobacteria, nitrite level was the most significant factor contributing to the total environmental effect. For Acidobacteria and Deinococcus-Thermus, this was ammonia and silicate levels, respectively (Table 4).

**Table 4:** Significant contributions to observed variation for bacterial phyla found in the rhizobiome.

Phylum	Source of variation			
	
	Unexplained	Environment^∗^	Contributing environmental factor	p-value
*Actinobacteria*	89.34%	10.66%	Nitrite	0.0103
*Acidobacteria*	95.32%	4.68%	Ammonia	0.0039
*Deinococcus-thermus*	97.22%	2.78%	Silicate	0.0372
*Proteobacteria*	63.96%	36.04%	Nitrite	0.0487


*^∗^combination of all environmental variables (geography, nutrient levels and temperature).*

**FIGURE 5:**
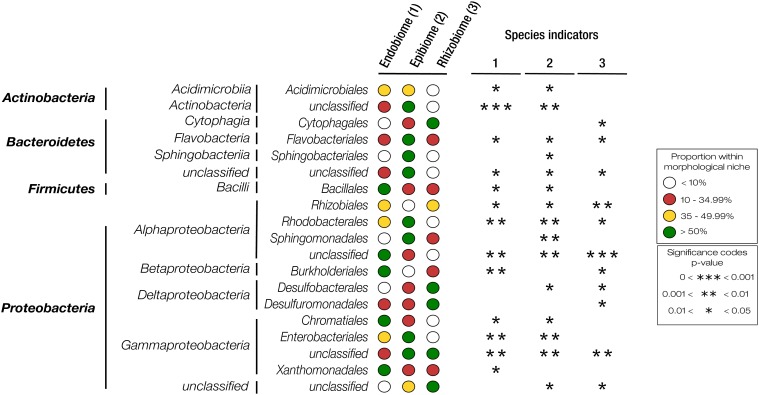
Results of the species indicator analysis and the analysis of similarity of variance between the three morphological niches. ANOSIM: *R* = 0.236; *P*-value = 0.0001. The proportion of each bacterial group within a morphological niche was calculated as a percentage of total reads assigned to each classification level.

Additionally, a species indicator analysis revealed certain bacteria responsible for the differences seen between each morphological niche (Figure 5). The majority of bacteria having a significant contribution to the variation seen belong to the phylum Proteobacteria, with unclassified Alphaproteobacteria, Rhizobiales, Rhodobacterales, and unclassified Gammaproteobacteria identified as significant indicator species for all three morphological niches. Bacteroidetes also contained species significant for all morphological niches, classified as Flavobacteriales and an unknown order. Xanthomonadales were exclusively significant for the endobiome, whereas Sphingobacteriales and Sphingomonadales were only significant in the epibiome. Cytophagales and Desulfuromonadales were significant indicator species for the rhizobiome. Indicator species exclusive for a particular niche correlated with high abundances (>50%) represented within the respective niches. For the endo- and epibiome, Acidimicrobiia, an unknown order of Actinobacteria, Chromatiales, and Enterobacteriales, were significant indicators, correspondingly found in low abundances (<10%) within the rhizobiome. Burkholderiales was the only order significantly shared by the endo- and rhizobiome, with low abundances in the epibiome.

## Discussion

This study characterizes bacterial communities associated to both native and invasive species of the green alga *Caulerpa* and investigates the taxonomic composition of these communities distributed on a macroscale between different geographical locations and at a microscale relating to location within an algal individual. Drivers influencing the observed bacterial community variation were assessed and partitioned into two categories describing environmental effects and the impact of morphological niche. Both the native and invasive species of *Caulerpa* are host to a rich microbiome, with unique OTUs specific to each species. Distinct communities were also associated with the different niches studied. While the majority of the variation observed remains unexplained, the largest identified portion (approx. 12%) was attributed to microscale location within the alga, i.e., the morphological niche. Approximately 8% of the variation was linked to a set of environmental parameters which was comprised of host species, biogeography, and nutrient profiles.

Bacterial variation has been shown to be influenced by several factors, including host species, geography and environmental parameters, with a large fraction remaining unexplained (Hollants et al., 2013). The results shown here in which the 78,5% of the observed microbial variation remains unexplained, are congruent with previous studies. A possible explanation for the high level of unexplained variation is that using the 16S rDNA gene meta-barcoding approach is insufficient to capture phenotypic differences between bacterial groups. This can be a result of a phylogenetic decoupling between the 16S rDNA gene and other functional capacities described for bacteria, assigning the same bacterial classification to functionally distinct bacteria. Alternatively, it could be a similarity threshold issue concerning the resolution of 16S rDNA gene binning based on limited reference databases or an overestimation of diversity due to intra-genomic heterogeneities of the 16S rRNA gene (Bodilis et al., 2012). Additionally the unexplained proportion can also be attributed to current variation models failing to simulate accurate environmental and spatial factors and therefore these models can only be used as exploratory tools (Gilbert and Bennett, 2010).

The bacterial variation observed suggests two main theories regarding the underlying mechanisms of bacterial recruitment. Firstly, that bacterial recruitment is predominantly deterministic and the high degree of variation could possibly be explained by environmental and biotic factors, or a complex combination thereof, which were not captured in this study. Indeed many physical and chemical properties of the surrounding seawater were not addressed in this study. While incorporating more factors into the analyses may resolve some of the unknown bacterial variation, it is likely a large proportion would remain unexplained as is observed in other studies (Hollants et al., 2013; Lima-Mendez et al., 2018). A second theory regarding bacterial recruitment is that the mechanisms involved are more stochastic in nature, but still adhere to certain deterministic elements. These elements drive bacterial recruitment and are reliant on functional organization of bacterial communities rather than the taxonomic component. Specific bacterial compositions therefore emerge, forming functionally stable interactions with their respective host (Fan et al., 2012). This concept is developed in context of algal-bacterial associations by the competitive lottery hypothesis proposed by Burke et al. (2011a), in which *Ulva* associated bacterial communities displayed a higher similarity based on metabolic capability rather than the taxonomic composition. Variation in bacterial communities between identical host species also indicates a high degree of stochasticity involved in bacterial recruitment dynamics (Bashan et al., 2016), suggesting that bacterial assembly involves a complex combination of deterministic and stochastic features (Zhou et al., 2013).

Variation between samples also could be attributed to temporal fluctuations of environmental factors, resulting in temporal bacterial variation as seen with the epibacterial communities of *Cystoseira compressa* (Mancuso et al., 2016). Moreover the influence of deterministic and stochastic factors have been found to be dynamic, affecting microbial community composition differently in relation to time (Zhou et al., 2014). Therefore different conditions present during algal thallus development could influence early microbial colonizers, resulting in alternative bacterial communities for morphological niches at different vegetative stages. Due to this added complexity, the developmental stage of the algal thallus combined with past environmental conditions should be taken into consideration when analyzing natural seaweed-bacterial communities. Assessing the true proportion of stochasticity involved in bacterial assembly within *Caulerpa* species would require monitoring associated microbial communities over time with a known starting microbiome. This could only be achieved through the initial establishment of axenic cultures or cultures with a predefined microbiome uniform across several individuals, which has not yet been developed for this genus.

Another factor to consider is host mediated microbial gardening through the release of chemical compounds (Kessler et al., 2018). Metabolic cooperation is essential in shaping microbial community structures (Mee et al., 2014), with complementary functions being facilitated by different bacteria that support holobiont stability and ecological fitness (Lemanceau et al., 2017). However, many bacteria perform similar functions leading to functional redundancy within bacterial populations (Yin et al., 2000). With complex bacterial interactions present within communities, this establishes a stochastic mechanism for recruitment of bacteria. The absence or presence of certain bacterial groups within a community is therefore dictated by inter-species interactions with existing bacterial groups and a degree of randomness caused by competition of functionally equivalent alternatives (Song et al., 2015; Bashan et al., 2016). The bacterial community that is initially associated to a host therefore partially governs the subsequent bacterial community succession.

The concept of functional priority in bacterial recruitment is further supported by the identification of unique communities associated to individual morphological niche. Differences in functional requirements for each microenvironment are evident in the literature, therefore variations in bacterial community composition on a microscale are expected (Stubbendieck et al., 2016). Studies done on *Sargassum muticum* also indicate a separation of distinct bacterial communities at a tissue level, and demonstrate that these communities respond differently to temporal factors (Serebryakova et al., 2018). Within *Caulerpa taxifolia*, distinct transcriptomic differences are identified between the base and apical region (Ranjan et al., 2015). Functional expression of genes is therefore niche-specific and bacterial association would follow suit. The epi- and endobiome communities of *Caulerpa* species have been shown to be distinct from one another (Aires et al., 2013). Epiphytic biofilms are complex systems that have been underestimated regarding their ecological impact and have been shown to provide essential metabolites for the ecological success of the host (Wahl et al., 2012; Egan et al., 2013). Endophytic symbionts have been shown to be tightly associated to their host and stable over time (Hollants et al., 2011; Arnaud-Haond et al., 2017). While it is assumed that epibacterial communities are less stable than endobionts due to increased susceptibility to environmental factors (Arnaud-Haond et al., 2017). The data shown in this study does not support this notion and demonstrates that the principle of stochastic recruitment is evident across both niches.

Factors influencing bacterial community composition in the rhizobiome can be subdivided into two levels; function-related and environment-related. Rhizoids of *Caulerpa* are involved in substrate adhesion as well as specialized functions such as nutrient uptake from the sediment and organic matter turnover (Chisholm and Moulin, 2003; Fagerberg et al., 2012). Surface bacteria, as well as intracellular bacteria have been identified within rhizoid structures of *Caulerpa taxifolia* and have been linked to metabolic functions such as nitrogen fixation (Chisholm et al., 1996). Therefore, the *Caulerpa* rhizobacterial community is distinct from other algal niches, reflecting the functional requirements of the rhizobiome. Additionally, marine sediments exhibit a horizontal heterogeneity that results in spatial variability of bacterial communities even on a centimeter scale (Scala and Kerkhof, 2000). In this study environmental factors are shown to have higher influence on the community composition of the rhizobiome than other morphological niches, with nitrate concentration contributing significantly to the variation observed. Furthermore sediment microbes have also been shown to influence the invasive potential of macrophytes, signifying that microbial community functions are important for successful invasions (Gribben et al., 2017). Comparisons between bacterial communities between native and invasive species of *Caulerpa*, have suggested that bacterial symbionts influence the invasive capacity (Arnaud-Haond et al., 2017). However, our data does not indicate that the invasive *C. cylindracea* has a bacterial advantage, based on the taxonomic structure of the communities. Additional investigations into the functional profiles in responses to different environments for both native and invasive species should be done to further elucidate the potential role microbes play in algal invasions.

Shifts in bacterial diversity over time and functional redundancy within bacterial groups allow for the continued stability of the algal holobiont. While exclusive bacterial interactions may be metabolically more efficient, there is an increased chance of ecological collapse. Therefore highly competitive bacterial groups promote flexible metabolic dependencies that sustain ecological stability (Coyte et al., 2015). Additionally the physiological state of a host influences bacterial colonization (Mancuso et al., 2016). Host physiology can be affected by the presence of parasitic organisms as well as environmental conditions. Unfavorable conditions may lead to the ecological demise of a host and introduce opportunistic bacterial colonizers (Gachon et al., 2010). Alternatively in such situations the host may attract bacteria able to alleviate stress effects.

The results presented in this study show that OTUs shared by both species tend to also be shared across morphological niches, whereas the majority of the species-specific OTUs are also unique to a specific niche (Figure 3). Niche specificity at an OTU level supports the concept of functional differentiation between niches and that bacterial recruitment is influenced by function (Burke et al., 2011a), which should be further investigated to confirm. Nevertheless, the 325 shared OTUs may be indicative of a conserved core microbiome specific to *Caulerpa*. However, the presence of a core microbiome should be further analyzed through the characterisation of functional profiles rather than taxonomic identification due to functional redundancy exhibited in bacterial groups (Lemanceau et al., 2017).

## Conclusion

In conclusion, this study extends the framework of the research previously done in this field, with several interesting findings complementary to the work done by Aires et al. (2015). The results here further elaborate on the separation of morphological niches and incorporate variation partitioning to further understand the driving forces involved in bacterial recruitment. While a considerable amount of the variation seen within bacterial communities is largely unexplained, it can be determined that location within an alga, here defined as distinct morphological niches, has a larger influence on the variation observed than biogeography and environmental factors. However, it is important to note that bacterial variations could also be accounted for by factors not included in this study such as temporal changes, developmental stage of the algal thallus, etc. In essence this study identifies unique bacterial communities for each morphological niche, indicating that variation within an algal individual is greater than variation between individuals and therefore supports the concept that functional requirements are involved in dictating bacterial assembly.

## Author Contributions

KM and ODC designed the research. KM and LC performed the sampling. KM performed the molecular work and analyzed the data. All authors contributed to the writing of the manuscript.

## Conflict of Interest Statement

The authors declare that the research was conducted in the absence of any commercial or financial relationships that could be construed as a potential conflict of interest.

## Supplementary Material

Figure S1 Rarefaction curve of raw sequence data. Morphological niches indicated by colors, endobiome (dark red), epibiome (green), rhizobiome (blue). Rarefaction cut off indicated by the black line. **(A)** *C. cylindracea* and **(B)** *C. prolifera.*

Figure S2 NMDS plot showing the β-diversity based on Bray-Curtis dissimilarity of the samples for each *Caulerpa* species, *C. cylindracea* (circle) and *C. prolifera* (triangle). Morphological niches are represented by colors, endobiome (red), epibiome (green), rhizobiome (blue).
