# Supplementary figures and images for: Disentangling the Influence of Environment, Host Specificity and Thallus Differentiation on Bacterial Communities in Siphonous Green Seaweeds
Source: Front Microbiol. 2019 Apr 5;10:717. doi: 10.3389/fmicb.2019.00717 (PMC6460459; doi:10.3389/fmicb.2019.00717)

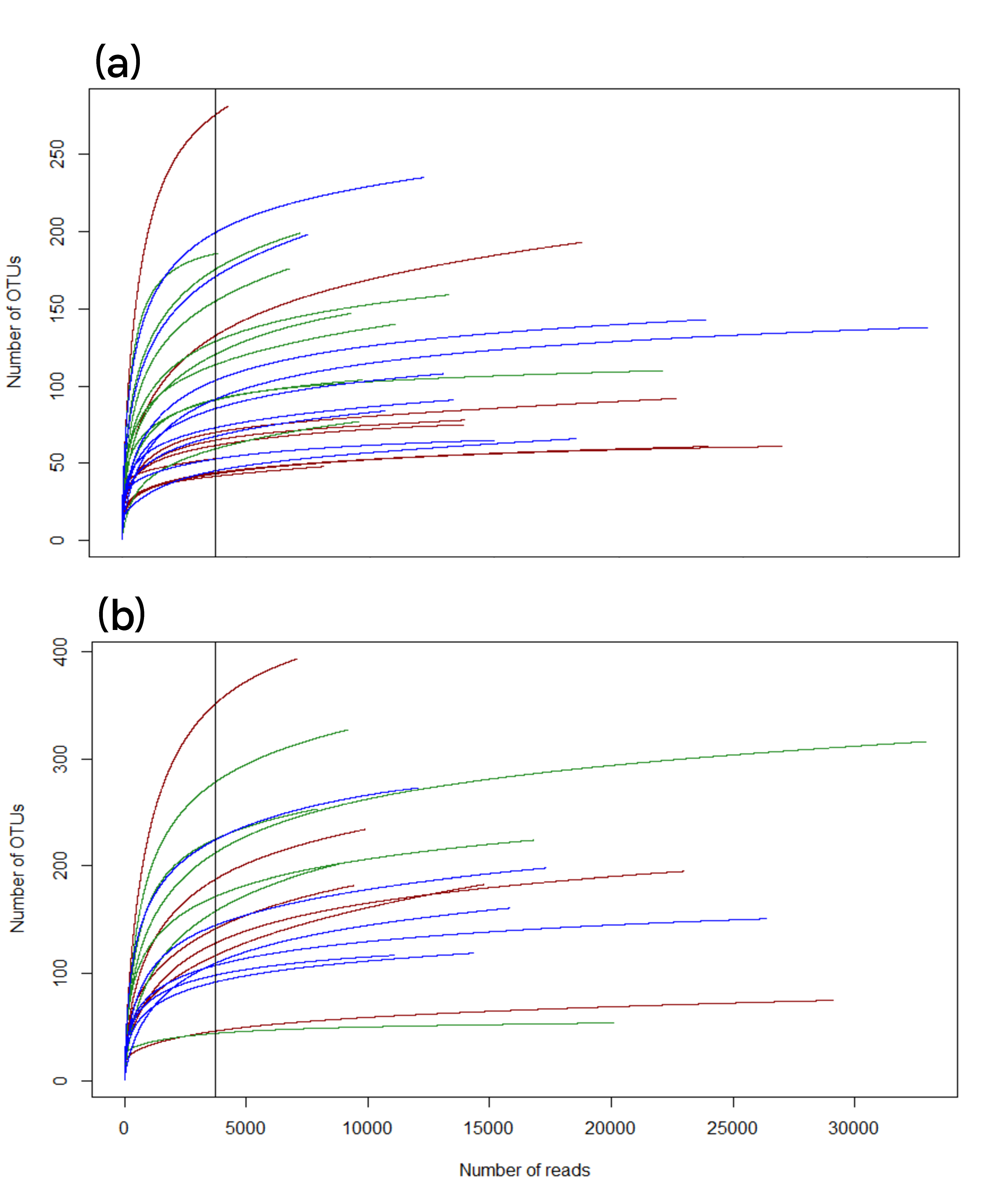

Supplement: Figure S1 — Rarefaction curve of raw sequence data. Morphological niches indicated by colors, endobiome (dark red), epibiome (green), rhizobiome (blue). Rarefaction cut off indicated by the black line. (A) C. cylindracea and (B) C. prolifera. [file Image_1.TIFF]

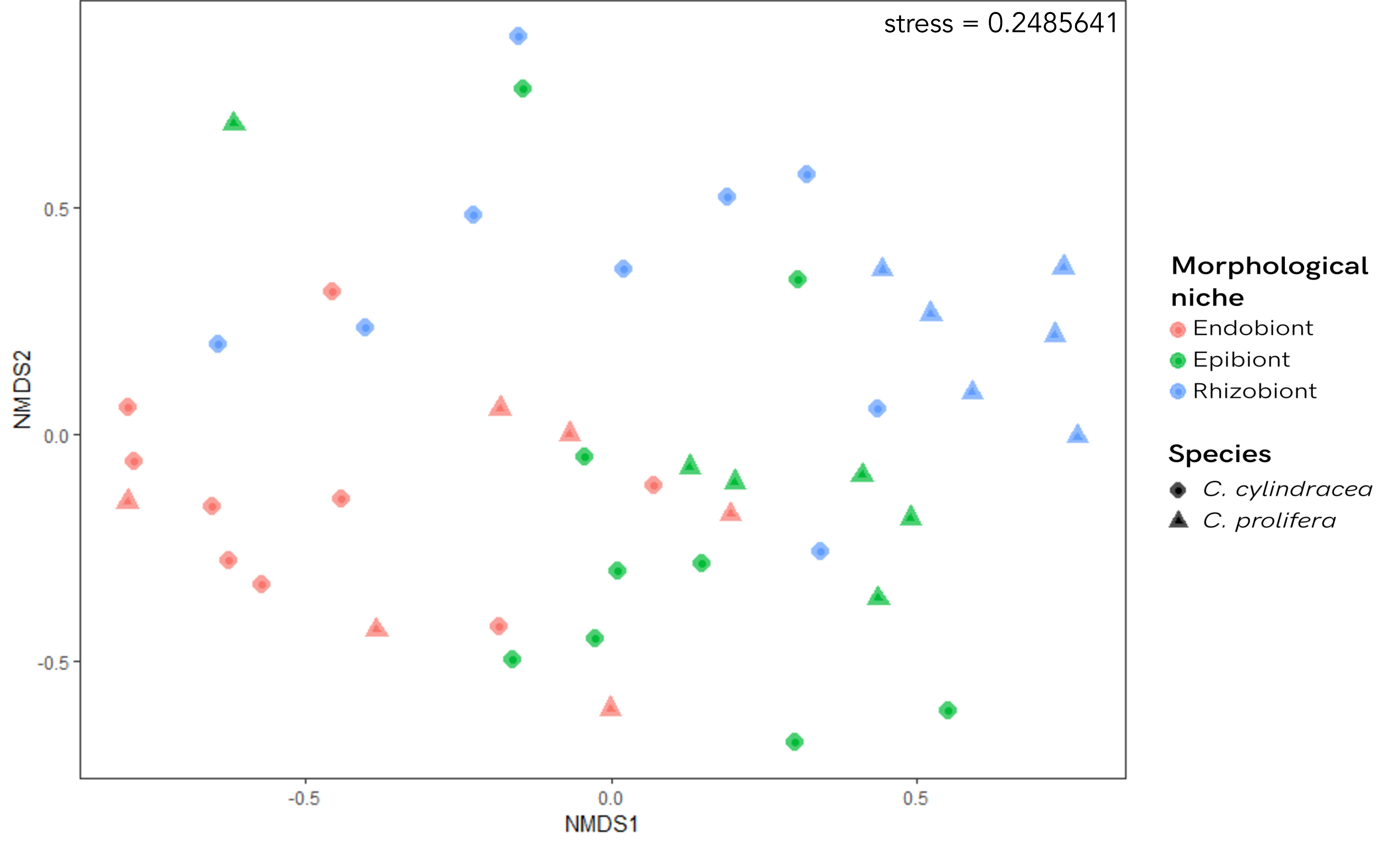

Supplement: Figure S2 — NMDS plot showing the β-diversity based on Bray-Curtis dissimilarity of the samples for each Caulerpa species, C. cylindracea (circle) and C. prolifera (triangle). Morphological niches are represented by colors, endobiome (red), epibiome (green), rhizobiome (blue). [file Image_2.TIFF]
