# Supplementary material for: Disentangling the Influence of Environment, Host Specificity and Thallus Differentiation on Bacterial Communities in Siphonous Green Seaweeds
Source: Front Microbiol. 2019 Apr 5;10:717. doi: 10.3389/fmicb.2019.00717 (PMC6460459; doi:10.3389/fmicb.2019.00717)
Supplement: Supplementary file 3 [file Table_1.DOCX]

| **Table S1. Overview of the samples** | | | | | | |
| --- | --- | --- | --- | --- | --- | --- |
| Sample ID | Type | Species of *Caulerpa* | Site | nr. Sequences | nr. OTUs | Good's Coverage (%) |
| c10 | rhizobionts | *cylindracea* | SF | 12941 | 109 | 99,81 |
| c11 | epibionts | *cylindracea* | SF | 13311 | 194 | 99,71 |
| c12 | endobionts | *cylindracea* | SF | 3640 | 65 | 99,67 |
| c13 | endobionts | *cylindracea* | SF | 14350 | 103 | 99,86 |
| c14 | rhizobionts | *cylindracea* | SF | 12252 | 299 | 99,63 |
| c15 | epibionts | *cylindracea* | SF | 11130 | 171 | 99,67 |
| c16 | endobionts | *cylindracea* | SF | 4219 | 315 | 98,51 |
| c17 | rhizobionts | *cylindracea* | SF | 14927 | 75 | 99,94 |
| c18 | epibionts | *cylindracea* | SF | 3835 | 206 | 99,40 |
| c19 | endobionts | *cylindracea* | DK | 21925 | 103 | 99,92 |
| c1 | rhizobionts | *cylindracea* | CS2 | 12959 | 128 | 99,75 |
| c20 | endobionts | *cylindracea* | DK | 7865 | 62 | 99,78 |
| c21 | epibionts | *cylindracea* | DK | 7019 | 253 | 99,27 |
| c22 | rhizobionts | *cylindracea* | DK | 7374 | 250 | 99,20 |
| c23 | endobionts | *cylindracea* | DK | 23130 | 71 | 99,96 |
| c24 | epibionts | *cylindracea* | DK | 9248 | 171 | 99,59 |
| c25 | rhizobionts | *cylindracea* | DK | 18181 | 90 | 99,87 |
| c26 | rhizobionts | *cylindracea* | DK | 10665 | 101 | 99,83 |
| c27 | epibionts | *cylindracea* | DK | 6697 | 222 | 99,22 |
| c2 | rhizobionts | *cylindracea* | CS2 | 23488 | 175 | 99,88 |
| c3 | rhizobionts | *cylindracea* | CS2 | 31944 | 172 | 99,91 |
| c4 | endobionts | *cylindracea* | CS2 | 17950 | 235 | 99,74 |
| c5 | epibionts | *cylindracea* | CS2 | 21674 | 133 | 99,93 |
| c6 | epibionts | *cylindracea* | CS2 | 9306 | 88 | 99,75 |
| c7 | endobionts | *cylindracea* | CS2 | 13082 | 95 | 99,89 |
| c8 | epibionts | *cylindracea* | CS2 | 9535 | 130 | 99,82 |
| c9 | endobionts | *cylindracea* | CS2 | 25976 | 71 | 99,95 |
| p19 | endobionts | *prolifera* | CS2 | 23691 | 238 | 99,81 |
| p20 | epibionts | *prolifera* | CS2 | 16753 | 245 | 99,80 |
| p21 | rhizobionts | *prolifera* | CS2 | 14695 | 141 | 99,84 |
| p22 | endobionts | *prolifera* | CS2 | 28498 | 86 | 99,94 |
| p23 | epibionts | *prolifera* | CS2 | 9106 | 363 | 99,31 |
| p24 | rhizobionts | *prolifera* | CS2 | 15532 | 197 | 99,67 |
| p25 | endobionts | *prolifera* | CS2 | 7120 | 469 | 98,65 |
| p26 | rhizobionts | *prolifera* | CS2 | 10956 | 148 | 99,77 |
| p27 | epibionts | *prolifera* | CS2 | 8849 | 224 | 99,42 |
| p58 | endobionts | *prolifera* | CS | 14208 | 216 | 99,53 |
| p59 | epibionts | *prolifera* | CS2 | 32547 | 390 | 99,81 |
| p60 | endobionts | *prolifera* | CS | 9660 | 277 | 99,39 |
| p61 | epibionts | *prolifera* | CS2 | 7902 | 292 | 99,41 |
| p62 | endobionts | *prolifera* | CS | 9747 | 210 | 99,34 |
| p63 | rhizobionts | *prolifera* | CS | 12208 | 355 | 99,52 |
| p64 | epibionts | *prolifera* | CS2 | 19504 | 70 | 99,94 |
| p65 | rhizobionts | *prolifera* | CS | 17406 | 259 | 99,71 |
| p66 | rhizobionts | *prolifera* | CS | 26233 | 209 | 99,83 |

| **Table S2.**  **PERMANOVA analyses based on Bray–Curtis dissimilarities of OTU abundances for bacterial communities within the whole microbiome and separate morphological niches.** | | | | | | |
| --- | --- | --- | --- | --- | --- | --- |
| Source | *df* | *SS* | *MS* | *Pseudo F* | *R²* | *p* |
| (a) Complete Microbiome |  |  |  |  |  |  |
| Morphological niche | 2 | 1,9385 | 0,96923 | 3,8935 | 0,13689 | **0,0001** |
| Host | 1 | 0,5079 | 0,5079 | 2,0403 | 0,03587 | **0,0222** |
| Environment | 3 | 1,6724 | 0,55748 | 2,2395 | 0,1181 | **0,0001** |
| Replicate number | 1 | 0,2232 | 0,2232 | 0,8966 | 0,01576 | 0,5362 |
| Morphological niche:Host | 2 | 0,7143 | 0,35713 | 1,4346 | 0,05044 | 0,0753 |
| Morphological niche:Environment | 6 | 2,1482 | 0,35803 | 1,4382 | 0,1517 | **0,0211** |
| Morphological niche:Replicate number | 2 | 0,4699 | 0,23495 | 0,9438 | 0,03318 | 0,5378 |
| Host:Replicate number | 1 | 0,2077 | 0,2077 | 0,8344 | 0,01467 | 0,6317 |
| Environment:Replicate number | 3 | 0,6364 | 0,21214 | 0,8522 | 0,04494 | 0,7418 |
| Morphological niche:Host:Replicate number | 2 | 0,4255 | 0,21277 | 0,8547 | 0,03005 | 0,6854 |
| Morphological niche:Environment:Replicate number | 6 | 1,4826 | 0,24711 | 0,9926 | 0,1047 | 0,5089 |
| Residuals | 15 | 3,734 | 0,24894 | 0,26369 |  |  |
| Total | 44 | 14,1607 | 1 |  |  |  |
| (b) Endobiome |  |  |  |  |  |  |
| Host | 1 | 0,3733 | 0,37331 | 1,7575 | 0,11444 | 0,0501 |
| Environment | 3 | 0,7705 | 0,25682 | 1,2091 | 0,23618 | 0,2074 |
| Replicate number | 1 | 0,1791 | 0,17906 | 0,843 | 0,05489 | 0,6327 |
| Host:Replicate number | 1 | 0,2371 | 0,2371 | 1,1162 | 0,07268 | 0,3014 |
| Environment:Replicate number | 3 | 0,6401 | 0,21338 | 1,0046 | 0,19624 | 0,4775 |
| Residuals | 5 | 1,0621 | 0,21241 | 0,32557 |  |  |
| Total | 14 | 3,2621 | 1 |  |  |  |
| (c) Epibiome |  |  |  |  |  |  |
| Host | 1 | 0,519 | 0,51903 | 1,92451 | 0,10584 | **0,037** |
| Environment | 3 | 1,6117 | 0,53722 | 1,99195 | 0,32866 | **0,0079** |
| Replicate number | 1 | 0,2905 | 0,29047 | 1,07703 | 0,05923 | 0,3657 |
| Host:Replicate number | 1 | 0,2593 | 0,25929 | 0,96141 | 0,05288 | 0,4781 |
| Environment:Replicate number | 3 | 0,8748 | 0,2916 | 1,08122 | 0,17839 | 0,378 |
| Residuals | 5 | 1,3485 | 0,26969 | 0,27499 |  |  |
| Total | 14 | 4,9037 | 1 |  |  |  |
| (d) Rhizobiome |  |  |  |  |  |  |
| Host | 1 | 0,3298 | 0,32982 | 1,24599 | 0,08131 | 0,2437 |
| Environment | 3 | 1,4385 | 0,4795 | 1,81147 | 0,35463 | **0,0431** |
| Replicate number | 1 | 0,2236 | 0,22357 | 0,84462 | 0,05512 | 0,568 |
| Host:Replicate number | 1 | 0,1368 | 0,13685 | 0,51699 | 0,03374 | 0,9006 |
| Environment:Replicate number | 3 | 0,6041 | 0,20137 | 0,76072 | 0,14893 | 0,7853 |
| Residuals | 5 | 1,3235 | 0,2647 | 0,32628 |  |  |
| Total | 14 | 4,0564 | 1 |  |  |  |
| The analysis was based on Bray–Curtis dissimilarities of OTU abundances and pseudo F statistics were calculated for each term using direct analogues to univariate expectations of mean squares (EMS); p-values were obtained using 9999 permutations under a reduced model; Significant p-values (alpha ≤ 0.05) are shown in bold. | | | | | | |
|  | | | | | | |
